# Strong Endemism of Bloom-Forming Tubular *Ulva* in Indian West Coast, with Description of *Ulva paschima* Sp. Nov. (Ulvales, Chlorophyta)

**DOI:** 10.1371/journal.pone.0109295

**Published:** 2014-10-15

**Authors:** Felix Bast, Aijaz Ahmad John, Satej Bhushan

**Affiliations:** Centre for Biosciences, Central University of Punjab, Bathinda, Punjab, India; University of Cambridge, United Kingdom

## Abstract

*Ulva intestinalis* and *Ulva compressa* are two bloom-forming morphologically-cryptic species of green seaweeds widely accepted as cosmopolitan in distribution. Previous studies have shown that these are two distinct species that exhibit great morphological plasticity with changing seawater salinity. Here we present a phylogeographic assessment of tubular *Ulva* that we considered belonging to this complex collected from various marine and estuarine green-tide occurrences in a ca. 600 km stretch of the Indian west coast. Maximum Likelihood and Bayesian Inference phylogenetic reconstructions using ITS nrDNA revealed strong endemism of Indian tubular *Ulva*, with none of the Indian isolates forming part of the already described phylogenetic clades of either *U. compressa* or *U. intestinalis*. Due to the straightforward conclusion that Indian isolates form a robust and distinct phylogenetic clade, a description of a new bloom-forming species, *Ulva paschima* Bast, is formally proposed. Our phylogenetic reconstructions using Neighbor-Joining method revealed evolutionary affinity of this new species with *Ulva flexuosa*. This is the first molecular assessment of *Ulva* from the Indian Subcontinent.

## Introduction

Genus *Ulva* (Linnaeus), commonly known as “Sea Lettuce”, encompasses some of the most ubiquitous green seaweeds distributed throughout the world, with habitats ranging from marine to freshwater. This algal genus is both beneficial and disadvantageous; beneficial as some species of this genus, including *Ulva prolifera* [1] and *Ulva intestinalis* [2], are commercially cultivated worldwide for its culinary use and disadvantageous as this genus is notorious for its ability to cause massive green-tides [3] and marine fouling [4]. Species of this genus are well known for having highly plastic morphologies, and that the habits can change from tube-form to blade-form or *vice versa* in response to changing environmental conditions [5]. Therefore, morphology-based classifications, which have been routinely used since the inception of this genus, are now being replaced with molecular systematics [6], [7]. For example, the genus *Enteromorpha*- which had been separated from *Ulva* based on tubular morphology by Link (Link in Nees 1820)- has recently been merged back to *Ulva* based on DNA sequence evidence [6]–[9]. Due to taxonomic confusions in morphology-based species delineation, concept of Operational Taxonomic Units (OTUs) have been used in recent phylogeographic assessments of *Ulva* from Hawaii [10] and USA [11].

Tubular *Ulva* in Indian Coast is believed to be comprised mainly of two species; *Ulva intestinalis* Linnaeus and *Ulva compressa* Linnaeus (personal observation). These two species are so closely related that they are regarded as cryptic species in a number of molecular phylogenetic studies [4], [12]. These species are separated from each other based on microscopic and macroscopic morphological characters [4]. Microscopic characters include distinct cell arrangement in *U. compressa* consisting of rosettes of cells (“Cell islands”) and longitudinal rows of cells in contrast to *U. intestinalis*, where there are no obvious arrangement of cells. Macroscopic characters include branching pattern and compression of thallus, in which *U. intestinalis* is mostly unbranched with hollow tubular monostromatic thalli, with very few branches for algae growing on low-saline environments, and *U. compressa* is highly branched with compressed thalli [13]. Taxonomic validity of these character states have been repeatedly questioned (see [4] for review). Morphological and phylogenetic variation in these two species has been investigated from the Baltic Sea Area [12] and the British Isles [4], and both of these reports concluded that *U. intestinalis* and *U. compressa* are distinct, monophyletic species. In recent phylogenetic assessments of *Ulva* from North Adriatic sea [14] and temperate Australia [8], [9] these two species together formed strongly supported clade, confirming their evolutionary relatedness.

The genus *Ulva* from India has never been subjected to extensive taxonomic scrutiny to date. While phylogeographic assessments of *Ulva* have been conducted in various parts of the world, including Japan [15], Australia [8], China [16], North-East Pacific [17] and Hawaii [16], sequence-based assessment of *Ulva* from Indian subcontinent have not yet been done. Objectives of the present study are to understand morphological and molecular variation of tubular *Ulva* occurring on the Indian west coast. Almost all of the previous phylogeographic assessments in genus *Ulva* were based on nucleoribosomal Internal Transcribed Spacer (ITS). ITS is one of the well-represented loci at Genbank and therefore we selected this locus for our molecular assessment.

## Materials and Methods

### Living Materials

During our 2012 expedition to the west coast of India, a particular tubular *Ulva* was detected causing massive blooms in a number of freely accessible locations (Table 1). Bloom specimens of tubular *Ulva*, either attached to intertidal substrates (including rocks, pebbles, wooden dinghies, mooring lines and breakwaters), or drifting while attached to a variety of floating objects were subsequently collected (Table 1). Collection coordinates were acquired with a handheld GPS device (eTrex 30, Garmin, USA). A map overlay of sampling locations with an accuracy of ± 10 meters is accessible at http://bit.ly/UlvaBloom. Photographs of the bloom were taken using a GPS-enabled digital camera (CyberShot DSC HX20V, Sony, Japan) and these photographs, with embedded GPS data, are available as online-only supplementary data (Figs. S1–10 in File S1). Seawater salinity was measured at the collection locations using a handheld salinometer (PCTTestr 35, Eutech Instruments, Singapore). Collected specimens were transported to the laboratory in zip-lock polythene bags under cold conditions (4–10°C). After washing the thalli in tap water to remove sediments and other contaminants, morphological characterization of the specimens was made using an upright microscope (BX53, Olympus, Japan) with an attached digital camera (E450, Olympus, Japan). Public domain software ImageJ (http://rsbweb.nih.gov/ij/) was used for scale calibration and size measurements. Pressed vouchers were prepared and deposited in the Central National Herbarium, Botanical Survey of India, Calcutta (*Index Herbariorum* code: CAL). Samples for molecular analyses were stored at -80°C awaiting further analysis.

**Table 1:** Collected samples of tubular *Ulva* from algal bloom across West Coast of India.

Location (administrative state in parenthesis) and Isolate identifier	Morphospecies	Genbank accession #	CAL voucher accession #	Habitat	Salinity PSU	Cell size in µm^2^ Mean±SD, n = 20	Coordinates
Anjuna (Goa)-ANJ	*U. intestinalis*	KF385504	CAL-CUPVOUCHER-UI-2013-3	Attached, Exposed rocky shore	34	142.81±2.80	15.58419N, 73.73683E
Karwar (Karnataka)-KAR	*U. intestinalis*	KF385502	CAL-CUPVOUCHER-UI-2013-2	Drifted, Exposed rocky shore	35	77.59±1.75	14.8064N, 74.1174E
Kundapur (Karnataka)-KUN	*U. compressa*	KF385505	CAL-CUPVOUCHER-UC-2013-1	Drifted, Sheltered inlet	24	97.65±3.14	13.63804N, 74.68797E
Mangalore (Karnataka)-MAN	*U. intestinalis*	KF385506	CAL-CUPVOUCHER-UI-2013-3a	Attached, Sheltered river-mouth	30	133.18±3.90	12.84831N, 74.82924E
Kannur (Kerala)-KAN	*U. intestinalis*	KF385503	CAL-CUPVOUCHER-UI-2013-4	Attached, Exposed rocky shore	33	97.28±6.17	11.853439N, 75.376644E
Ponnani (Kerala)-PON	*U. intestinalis*	KF385501	CAL-CUPVOUCHER-UI-2013-5	Attached, Exposed rocky shore	31	52.19±3.23	10.78637N, 75.91265E

### DNA extraction, PCR amplification, purification and DNA sequencing

The frozen specimens were thawed in artificial sea water [18]. Total genomic DNA was extracted from the specimens using a HiPurA Algal Genomic Extraction Kit (HiMedia Laboratories Pvt. Ltd., Mumbai) following manufacturer's protocol. Tissues from the apical thalli were selected to increase DNA yield. The quality of DNA was checked on 0.8% agarose gel and the quantity of DNA was checked with spectrophotometer. Isolated DNA was stored at −20°C.

### PCR amplification

A DNA working solution of 25 ng/µl was prepared for polymerase chain reaction (PCR) in a separate tube. The 20 µl PCR reaction mix contained 2 µl of 10× reaction buffer with 15 mM MgCl_2_ (Applied Biosystems, India), 4 µl each of 10 µM primer, 2 µl of 1 µM dNTPs (Imperial Life sciences, India), 0.6 unit of rTaq DNA polymerase (Imperial Life sciences, India), 4 µl of template DNA and sterile water. The four universal primers used for amplifying the ITS regions and the 5.8S gene (fragment length = 639 bp) were: ITS1 (5'-TCCGTAGGTGAACCTGCGG-3'), ITS2 (5'- GCTGCGTTCTTCATCGATGC-3'), ITS3 (5'- GCATCGATGAAGAACGCAGC-3') and ITS4 (5'- TCCTCCGCTTATTGATATGC-3') [19]. PCR amplifications were carried out in programmable thermal cycler (Veriti, ABI, USA) and reaction profile included an initial denaturation at 94°C for 5 minutes, followed by 35 cycles of 94°C for 1 minute, 52°C for 2 minutes and 72°C for 2 minutes, and a final extension of 72°C for 10 minutes.

### Purification of PCR product

Amplicons were purified using ExoSAP-IT PCR clean-up kit following manufacturer's instructions (USB Corporation, Cleveland, OH, USA). A working solution of 1∶10 (DNA: water) was prepared as sequencing template. PCR amplification reactions (as well as its sequencing) were carried out in duplicate for each target sequence of each isolate using the same set of primers as a quality control.

### DNA sequencing

Purified PCR products were subjected to bidirectional Sanger sequencing using a dideoxy chain termination protocol with ABI BigDye Terminator Cycle Sequencing Ready Reaction Kit v3.1 (Applied Biosystems, Foster City, CA, USA) and a programmable thermal cycler (Veriti, ABI, USA), as per [20].DNA sequences were assembled using the computer program CodonCodeAligner (CodonCode Corporation, USA). Sequences were deposited in Genbank (Table 1).

### Phylogenetic analysis

We followed the step-by-step protocol for phylogenetic analysis, including alignment construction, Maximum Likelihood test to find best-fitting substitution models [21], phylogeny reconstruction using Maximum Likelihood (ML), Bayesian Inference (BI) and distance analysis as outlined in Bast [22]. In summary, six sequences of tubular *Ulva* from India were aligned with other published accessions *of Ulva intestinalis* and *Ulva compressa* obtained from Genbank (Table S1) by MUSCLE algorithm inside computer program Geneious v6.1.6 (available at http://www.genious.com) and alignments were edited by eye. Phylogenetic analysis using ML algorithm was conducted in MEGA (www.megasoftware.net/) with starting tree generated by BioNJ. Substitution bias was modelled by Tamura-3-Parameter [23] (T3P) model with Gamma distribution (that was the best model in our test to find best fitting substitution models [21] with BIC (Bayesian Information Criterion) score of 5626.537).Heuristic searches were performed with tree bisection-reconnection, MULTREES and steepest descent options in effect. 1000 bootstrap replicates were performed under ML criterion to estimate interior branch support [24]. Phylogenetic analysis with BI was conducted using the MrBayes plug-in v3 [25] within Geneious. Analyses were run with four Markov chains using the T3P model with Gamma distribution for 10^6^ generations with a tree saved every 100^th^ generation. The first 1000 trees were discarded as burn-in, as determined by “burnin <number>“ function of MrBayes plug-in. A consensus tree was constructed using the consensus tree builder within Geneious. In order to investigate relative phylogenetic position of our isolates in genus *Ulva*, a separate ITS dataset was constructed with 120 sequences obtained from Genbank, spanning all major species represented in the database. Due to the computational limitations, we used Neighbor-Joining (NJ) method for this dataset. All of our scientific datasets, including cell area measurements, DNA sequence alignment in FASTA format, results of ModelTest, T3P pairwise distances, tree in nexus format and original electropherograms of DNA sequences with contig assembly instructions are freely available at LabArchives (http://dx.doi.org/10.6070/H4639MP5).

### Nomenclature

The electronic version of this article in Portable Document Format (PDF) in a work with an ISSN or ISBN will represent a published work according to the International Code of Nomenclature for algae, fungi, and plants, and hence the new names contained in the electronic publication of a PLOS ONE article are effectively published under that Code from the electronic edition alone, so there is no longer any need to provide printed copies. The online version of this work is archived and available from the following digital repositories: PubMed Central, LOCKSS and Research Gate.

## Results

**Figure 1:**
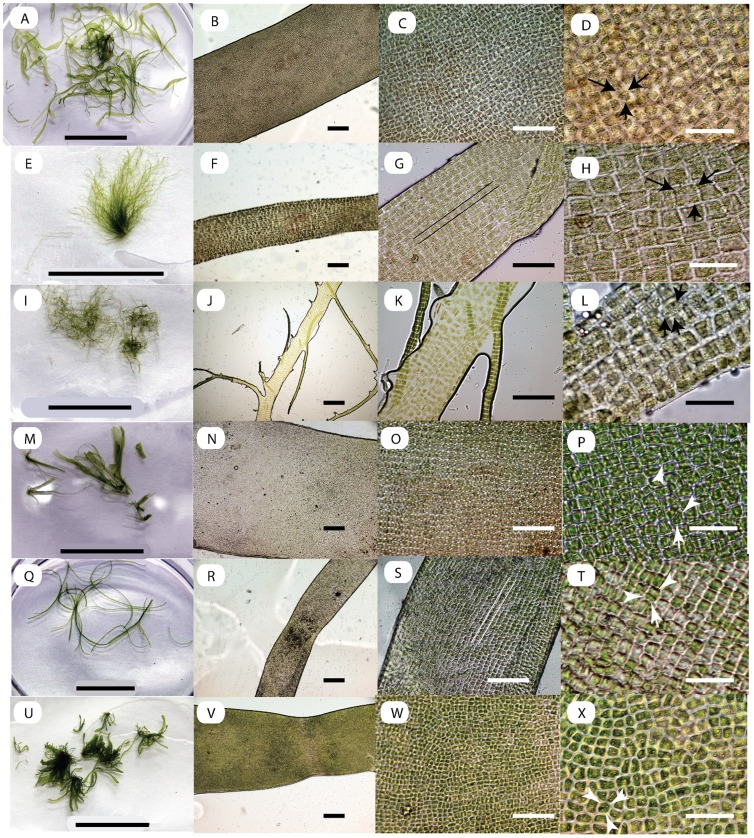
Morphology of tubular *Ulva* from India. A–D ANJ Isolate, E–H KAR isolate, I–L KUN isolate, M-P MAN isolate, Q–T KAN Isolate and U–X PON Isolate. *Arrowheads* in M and U indicate flat portions of thalli, J indicate branching pattern and D, H, L, P, T and X indicate pyrenoids. Scale bars are 2 mm for A, E, I, M, Q and U; 200 µm for B, F, J, N, R and V; 100 µm for C, G, K, O, S and W; and 50 µm for D, H, L, P, T and X.

Seawater salinity ranged between 35PSU and 24PSU. As expected, exposed shores had higher salinity than inlets and river mouth areas. On external morphology, all six isolates had their own unique features (Fig 1, and Figs S1–S10 in File S1). All isolates were grass green in color, erect filamentous, and had a parietal chloroplast with more than three pyrenoids inside each cell (*Arrowheads* in Figs 1 D, H, L, P, T and X). Isolates ANJ, MAN and PON had some part of their thallus flattened (indicated by *Arrowheads* in Figs 1A, M and U, respectively) and had thicker thalli (Figs 1B, N and V), comparing with other isolates. In terms of thalli branching character state, isolate KUN was unique in that it was branched, up to two lateral branch orders (*Arrowheads* in Fig 1J), while the other isolates were unbranched. In terms of cell arrangement, isolates KAR and KAN were similar, with more or less linear arrangement of cells (cell layers accentuated with pairs of lines in Figs 1G and S, respectively). In terms of cell size, isolates ANJ and MAN were the largest and PON was the smallest (Table 1). A comparison of taxonomically relevant morphological characters for our isolates with *U. intestinalis*, *U. compressa* and *U. flexuosa* is presented (Table 2). As per thallus branching character and thallus compression character, isolates ANJ, KAR, MAN, KAN and PON were arbitrarily classified as *U. intestinalis* (unbranched, hollow) and isolate KUN as *Ulva compressa* (branched, compressed).

**Table 2:** Morphological characters of Indian isolates in comparison with *Ulva intestinalis*, *Ulva compressa* and *Ulva flexuosa* [29], [30].

Character	Isolates		*Ulva intestinalis*	*Ulva compressa*	*Ulva flexuosa*
	ANJ, KAR, MAN, KAN, PON	KUN			
Tubular thallus branched or unbranched	Unbranched	Branched	Mostly unbranched	Branched	Mostly branched
Tubular thallus hollow or compressed	Hollow	Compressed	Hollow	Compressed	Hollow/Compressed
Cell arrangement: Linear or Nonlinear	Linear (Only for KAR and KAN)	Nonlinear	Nonlinear	Linear	Linear
Cell arrangement: Rosettes	Absent	Absent	Absent	Present	Absent

**Figure 2:**
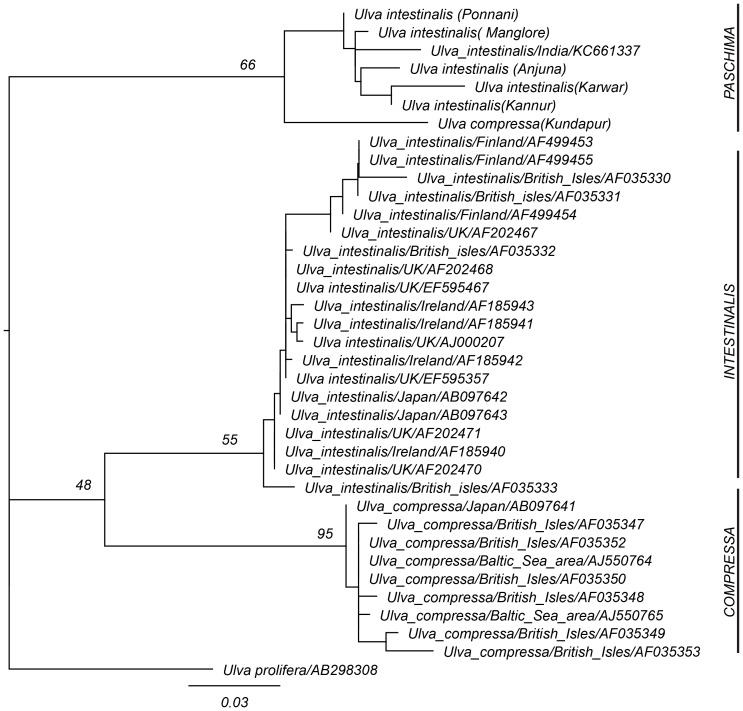
Phylogenetic position of tubular *Ulva* isolates from India among other tubular *Ulva* accessions in ITS dataset using Maximum Likelihood phylogenetic reconstruction (LnL = −2412.46) with T3P model of molecular evolution with gamma distribution (T3P+G). Numbers near nodes represent bootstrap support (1000 replicates), exceeding 50. This phylogram is rooted with *Ulva prolifera* as outgroup. Scale bar given on bottom is in the units of average nucleotide substitutions per site.

**Figure 3:**
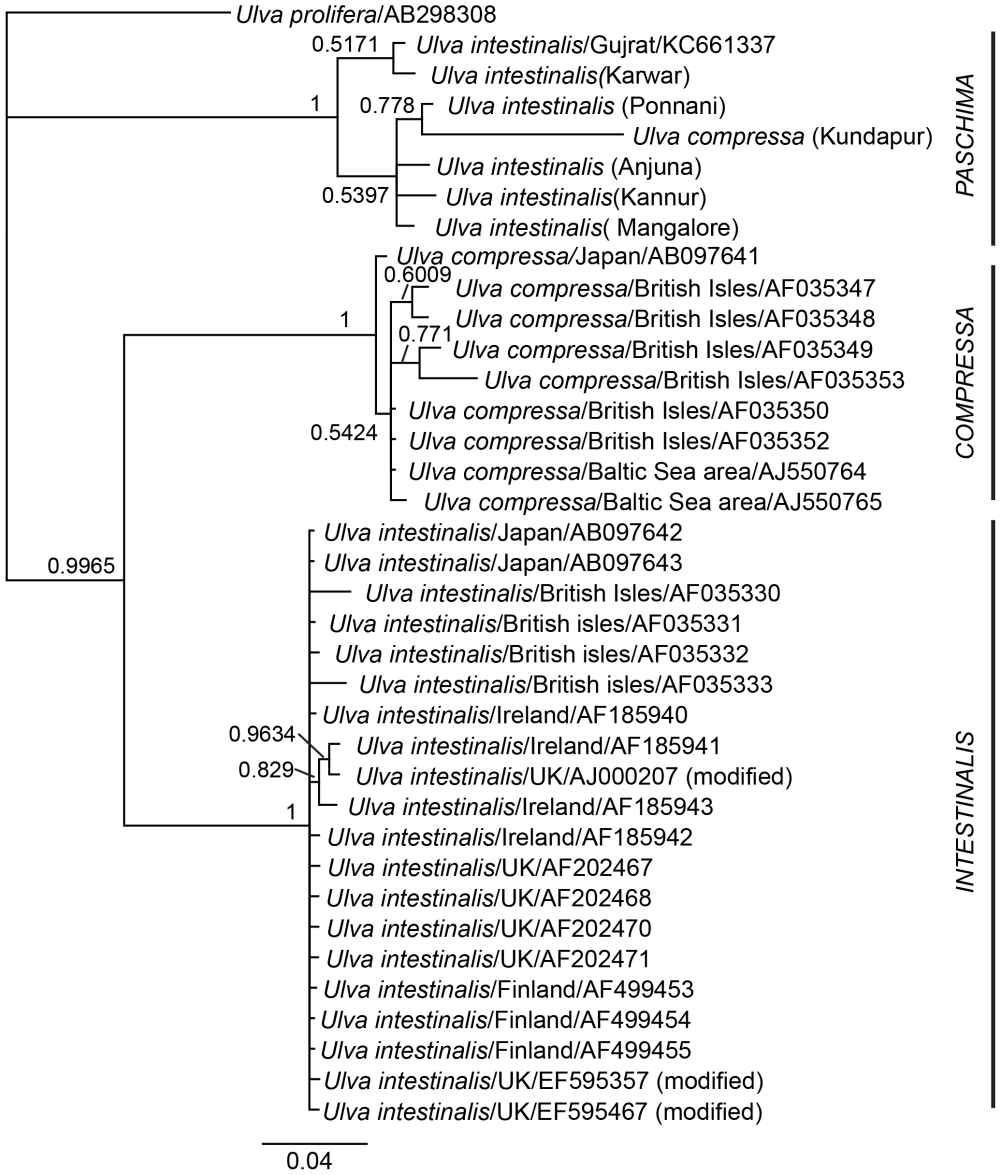
Phylogenetic position of tubular *Ulva* isolates from India among other tubular *Ulva* accessions in ITS dataset using Bayesian Inference phylogenetic reconstruction (LnL = −2628.193) with T3P model of molecular evolution with gamma distribution (T3P+G). Numbers near nodes represent Bayesian Posterior Probabilities, exceeding 0.5. This phylogram is rooted with *Ulva prolifera* as outgroup. Scale bar given on bottom is in the units of average nucleotide substitutions per site.

While we primarily employed ITS sequence data for barcoding, phylogenetic reconstruction using this locus revealed a number of evolutionary trends. Phylogenetic reconstruction using the ML (Fig 2) and BI (Fig 3) methods resulted in moderately-resolved phylograms, with three clades. All Indian isolates of tubular *Ulva* formed a single clade (highlighted “Paschima”). Our isolate KUN (*U. compressa* morphotype) seems to have been much diverged from Indian isolates of *U. intestinalis* morphotype as evidenced by long branch-length. This isolate clustered within *U. intestinalis* accessions from India in BI, but was basal to *U. intestinalis* accessions from India in ML. Other monophyletic clades included that of non-Indian isolates of *U. compressa* (highlighted “Compressa”) and *U. intestinalis* (highlighted “Intestinalis). Within-group mean T3P distance was 0.5352 for “Paschima” and 0.000 for both “Intestinalis” and “Compressa”, which indicates a very high genetic heterogeneity for the “Paschima” clade. In one study [26], within-group JC (Jukes-Cantor) distance for *Enteromorpha* had been reported to range between 0.09 to 0.16. As within- group distance for “Paschima” clade observed in the present study being much higher, possibility that our isolate KUN belonging to another unique taxon from India cannot be ruled out.

**Figure 4:**
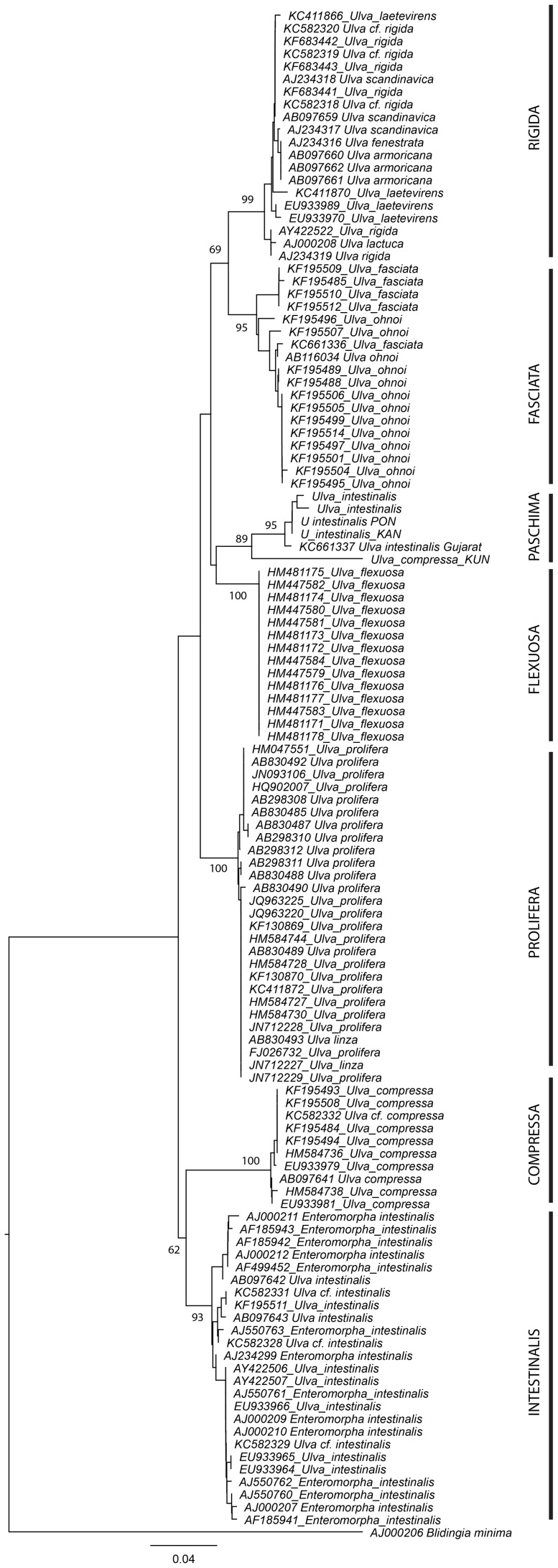
Phylogenetic position of tubular *Ulva* isolates from India among other accessions in ITS dataset using Neighbor-Joining Inference phylogenetic reconstruction (total tree length =  0.79153595) with T3P model of molecular evolution with gamma distribution (T3P+G). Numbers near nodes represent bootstrap support (100 replicates), exceeding 50. This phylogram is rooted with *Blidingia minima* as outgroup. Scale bar given on bottom is in the units of average nucleotide substitutions per site.

Phylogenetic analysis with NJ conducted for 121 sequences of *Ulva* resulted in a moderately-resolved phylogram (Fig.4). Paschima clade showed evolutionary affinity to Flexuosa clade, albeit with weak bootstrap support (36, not shown in figure). *Ulva flexuosa*, *Ulva compressa*, and *Ulva intestinalis* formed respective monophyletic clades. A clade comprising of *Ulva rigida*, *Ulva laetevirens*, *Ulva scandinavica*, *Ulva fenestrata*, *Ulva armoricana* and *Ulva lactuca* had strong bootstrap support, indicating phylogenetic affinity of these species. *Ulva fasciata* formed a strongly supported clade with *Ulva ohnoi* (clade “fasciata”) and *Ulva linza* clustered within a strongly supported clade comprising of *Ulva prolifera* (clade “prolifera”). For a definitive phylogenetic assessment of these taxa, additional genetic loci need to be employed.

## Discussion

The present study made several interesting revelations, the most significant of which is the apparent endemism of a bloom-forming Indian tubular *Ulva* that is morphologically plastic and indistinguishable from *U. intestinalis* and *U. compressa*. Contrary to our expectations, the five isolates that had hollow, unbranched thalli did not group within the already described *U. intestinalis* clade in our phylogenetic analyses, nor did the single identified isolate that had compressed, branched thalli show affiliation to the *U. compressa* clade. Instead, all of our isolates formed a strongly supported clade, which showed affinity to a previous sample identified as *Ulva intestinalis* from Gopnath, Gujarat, India (personal communication). This clearly indicates a high degree of endemism for the Indian tubular *Ulva*. Interestingly, Japanese isolates of either *Ulva compressa*, or *Ulva intestinalis*, were described to have very little pair-wise distance from European Isolates at ITS loci[27]. Given the vast geographical distance of ca. 10,000 km, this earlier report could either be suggestive of a recent introduction of these species to either of these locations or existence of temperate haplotypes. A recent report on the molecular assessment of *Ulva* from Australia concluded that the genus encompasses a number of endemic potentially cryptic species in addition to cosmopolitan species [8]. In the light of these findings, assumptions of cosmopolitanism among certain species of *Ulva* can cause novel and endemic species to be overlooked.

Our identification of the KUN isolate as *U. compressa* was based on the previously described character states of branching pattern of thallus and compressed state of the filament [4]. However, phylogeny reconstruction clustered our *U. compressa* specimen within a clade comprised chiefly of hollow, unbranched tubular *Ulva* from India (similar to *U. intestinalis* morphospecies). These two tubular *Ulva* morphospecies from India might indeed be conspecific. Low salinity at the habitat of the KUN isolate might have influenced the species to acquire this morphotype as suggested by previous studies [4]. Alternately, non-Indian accessions of *U. intestinalis* and *U. compressa* might indeed be unique species with yet-to-discover synapomorphic character state/s, as observed in our phylogenetic analyses. In summary, without molecular data, Indian species of bloom-forming *Ulva* most closely resemble with either/both *U. intestinalis* and *U. compressa*, two species that are shown in the literature to be difficult to distinguish between.

Results from our phylogenetic reconstructions strongly argue in favor of species-level taxonomic treatment for the OTUs from India, which is evolutionarily unrelated to either *Ulva intestinalis* or *Ulva compressa*. We therefore formally propose a new species of bloom-forming tubular *Ulva* as per the following description, congruent with Phylogenetic Species Concept [28]:

### *Ulva paschima* Bast sp. nov. (Fig 1)

#### Description

Primary diagnosis is the phylogenetic affiliation of OTUs with ITS clade “Paschima” as per this report. Fronds erect filamentous and grass green in color; 5 cm–40 cm in length; mostly unbranched tubular with some parts of the thalli compressed or flat, ribbon-like; tufts of filamentous thalli attached via rhizoid. Morphotype in low-saline inlets and estuaries might have branched, compressed thalli. Cells are more or less quadrilateral; some have linear cell arrangement. Parietal chloroplast with>2 pyrenoids per cell.

#### Holotype

Collected from intertidal rocks at a splash zone near Paraiso de Goa, Anjuna Beach, Goa, India (15.58419N, 73.73683E). Deposited at Central National Herbarium, Botanical Survey of India, Calcutta (*Index Herbariorum* code: CAL) under voucher # CAL-CUPVOUCHER-UP-2013-3. DNA sequences of nrDNA ITS1-5.8S-ITS2 complete region of the holotype deposited at Genbank under accession # KF385504.

#### Isotype

Deposited at Herbarium, the Central University of Punjab under voucher No.: CUPVOUCHER-UP-2013-3. Frozen voucher maintained at Centre for Biosciences, the Central University of Punjab under voucher No.: CUPFVOUCHER-UP-2013-1.

#### Etymology

Specific epithet in Sanskrit means “west” where the algae is first described in Indian Subcontinent.

## Supporting Information

File S1 **Compressed file containing Figure S1 to Figure S10. Figure S1,** Photograph of algal bloom specimen of *Ulva intestinalis* isolate ANJ. **Figure S2**, Photograph of drifting algal bloom specimen of *Ulva intestinalis* isolate KAR, attached on mooring line. **Figure S3**, Photograph of algal bloom specimen of *Ulva compressa* isolate KUN. **Figure S4**, Photograph of algal bloom specimen of *Ulva compressa* isolate KUN. **Figure S5**, Photograph of algal bloom specimen of *Ulva compressa* isolate KUN. **Figure S6**, Photograph of algal bloom specimen of *Ulva intestinalis* isolate MAN. **Figure S7**, Photograph of algal bloom specimen of *Ulva intestinalis* isolate MAN, attached on wooden dinghy. **Figure S8**, Photograph of algal bloom specimen of *Ulva intestinalis* isolate MAN, attached on wooden dinghy. **Figure S9**, Photograph of algal bloom specimen of *Ulva intestinalis* isolate KAN. **Figure S10**, Photograph of algal bloom specimen of *Ulva intestinalis* isolate PON.
(RAR)

Table S1 **Sequences of nuclear rDNA ITS regions procured from Genbank used in this study.**
(DOCX)
