# Supplementary material for: Strong Endemism of Bloom-Forming Tubular Ulva in Indian West Coast, with Description of Ulva paschima Sp. Nov. (Ulvales, Chlorophyta)
Source: PLoS One. 2014 Oct 15;9(10):e109295. doi: 10.1371/journal.pone.0109295 (PMC4198087; doi:10.1371/journal.pone.0109295)
Supplement: Table S1 — Sequences of nuclear rDNA ITS regions procured from Genbank used in this study. (DOCX) [file pone.0109295.s002.docx]

**Table.S1:** Sequences of nuclear rDNA ITS regions procured from Genbank used in this study

| **S. No.** | **Place of collection** | **Source/or reference** | **Accession No.** | **Species** |
| --- | --- | --- | --- | --- |
| 1 | India  (Gopnath, Bhavnavar, Gujrat) | Unpubl. | KC661337 | *Ulva intestinalis* |
| 2 | Japan  (Nagasaki, Teguma) | [[31](#_ENREF_31)] | AB097641 | *Ulva compressa* |
| 3 | Japan  (Hokkaido, Shimamaki) | [[31](#_ENREF_31)] | AB097642 | *Ulva intestinalis* |
| 4 | Japan  (Sweden: Karlskrona) | [[31](#_ENREF_31)] | AB097643 | *Ulva intestinalis* |
| 5 | British isles  (Rathlin Island, Co. Antrium) | [[4](#_ENREF_4)] | AF035330 | *Ulva intestinalis* |
| 6 | British isles  (Rathlin Island, Co. Antrium) | [[4](#_ENREF_4)] | AF035331 | *Ulva intestinalis* |
| 7 | British isles  (Solva, Pembroke, Wales) | [[4](#_ENREF_4)] | AF035332 | *Ulva intestinalis* |
| 8 | British isles  (Rathlin Island, Co. Antrium) | [[4](#_ENREF_4)] | AF035333 | *Ulva intestinalis* |
| 9 | Ireland  (Carnelea, Belfast Lough, N. Ireland) | [[32](#_ENREF_32)] | AF185940 | *Ulva intestinalis* |
| 10 | Ireland  (Fresh water stream, co. Waterford) | [[32](#_ENREF_32)] | AF185941 | *Ulva intestinalis* |
| 11 | Ireland  (Laganside, Belfast Lough, N. Ireland) | [[32](#_ENREF_32)] | AF185942 | *Ulva intestinalis* |
| 12 | Ireland  (Granegh Bay, Strangford lough, N. Ireland) | [[32](#_ENREF_32)] | AF185943 | *Ulva intestinalis* |
| 13 | UK  (Granagh Bay, Strangford Lough N. Ireland) | [[32](#_ENREF_32)] | AF202467 | *Ulva intestinalis* |
| 14 | UK  (Granagh Bay, Strangford Lough N. Ireland) | [[32](#_ENREF_32)] | AF202468 | *Ulva intestinalis* |
| 15 | UK  (Carnalea Belfast lough, N. Ireland) | [[32](#_ENREF_32)] | AF202470 | *Ulva intestinalis* |
| 16 | UK Carnalea (Belfast lough, N. Ireland) | [[32](#_ENREF_32)] | AF202471 | *Ulva intestinalis* |
| 17 | UK  (Yathan Estuary, Aberdeenshire Scotland) | [[5](#_ENREF_5)] | AJ000207 | *Ulva intestinalis* |
| 18 | UK  (West Sutherland, Talmine) | [[33](#_ENREF_33)] | EF595357 | *Ulva intestinalis* |
| 19 | UK  (West Sutherland, Talmine) | [[33](#_ENREF_33)] | EF595467 | *Ulva intestinalis* |
| 20 | Finland  (Hanko, Tvarminne) | [[27](#_ENREF_27)] | AF499453 | *Ulva intestinalis* |
| 21 | Finland  (Espoo, Haukilahti) | [[27](#_ENREF_27)] | AF499454 | *Ulva intestinalis* |
| 22 | Finland  (Espoo, Haukilahti) | [[27](#_ENREF_27)] | AF499455 | *Ulva intestinalis* |
| 23 | British isles  (Portaferry Strangford Lough) | [[4](#_ENREF_4)] | AF035347 | *Ulva compressa* |
| 24 | British isles  (Quarterland Bay Strangford Lough) | [[4](#_ENREF_4)] | AF035348 | *Ulva compressa* |
| 25 | British isles  (Belfast Harbour, Belfast Lough) | [[4](#_ENREF_4)] | AF035349 | *Ulva compressa* |
| 26 | British isles  (Portaferry Strangford Lough) | [[4](#_ENREF_4)] | AF035350 | *Ulva compressa* |
| 27 | British isles  (Coral Strand Greatman’s Bay) | [[4](#_ENREF_4)] | AF035352 | *Ulva compressa* |
| 28 | British isles  (Granagh Bay, Strangford Lough) | [[4](#_ENREF_4)] | AF035353 | *Ulva compressa* |
| 29 | Baltic Sea  (Sweden: Skagerrak) | [[12](#_ENREF_12)] | AJ550764 | *Ulva compressa* |
| 30 | Baltic Sea  (Netherlands: Westcapelle) | [[12](#_ENREF_12)] | AJ550765 | *Ulva compressa* |
